# Joint Analysis for Multivariate Longitudinal and Event Time Data With a Change Point Anchored at Interval‐Censored Event Time

**DOI:** 10.1002/sim.70765

**Published:** 2026-10-09

**Authors:** Yue Zhan, Cheng Zheng, Ying Zhang

**Affiliations:** ^1^ Department of Biostatistics, College of Public Health University of Nebraska Medical Center Omaha Nebraska USA

**Keywords:** change point, interval‐censored data, joint model, longitudinal biomarker, survival analysis

## Abstract

Huntington's disease (HD) is an autosomal dominant neurodegenerative disorder characterized by motor dysfunction, psychiatric disturbances, and cognitive decline. The onset of HD is marked by severe motor impairment, which may be predicted by prior cognitive decline and, in turn, exacerbate cognitive deficits. Clinical data, however, are often collected at discrete time points, so the timing of disease onset is subject to interval censoring. To address the challenges posed by such data, we develop a joint model for multivariate longitudinal biomarkers with a change point anchored at an interval‐censored event time. The model simultaneously assesses the effects of longitudinal biomarkers on the event time and the changes in biomarker trajectories following the event. We conduct a comprehensive simulation study to demonstrate the finite‐sample performance of the proposed method for statistical inference based on the asymptotic normality theorem. Finally, we apply the method to PREDICT‐HD, a multisite observational cohort study of prodromal HD individuals, to ascertain how cognitive impairment and motor dysfunction interact during disease progression.

## 1 Introduction

Huntington's disease (HD) is an inherited, autosomal dominant neurodegenerative disorder characterized by progressive motor dysfunction, cognitive decline, and psychiatric disturbances. The disease is caused by an abnormal expansion of CAG trinucleotide repeats in the *HTT* gene on chromosome 4, which leads to the production of a mutant huntingtin protein that disrupts normal cellular function [1, 2]. Currently, there is no effective treatment available for HD patients.

Huntington's disease encompasses motor, cognitive, and psychiatric manifestations, but the clinical diagnosis of onset is determined primarily by the emergence of motor symptoms [3]. However, cognitive impairment has been found to appear prior to HD motor diagnosis [4, 5, 6, 7, 8]. Zhang et al. [8] have thoroughly studied mild cognitive impairment (MCI) in various cognitive domains characterized by Harrington et al. [9], which is regarded as an early landmark for disease progression in prodromal HD individuals. Although rapid cognitive decline has been observed during the early stages of HD [10], a comprehensive investigation of how cognitive impairment interacts with HD onset is lacking. Such research could provide important insights into disease progression in individuals with prodromal HD. In this work, we propose a holistic model to uncover the relationship between cognitive decline in multiple domains and HD onset using joint modeling of multivariate longitudinal and event time data.

Joint modeling of longitudinal and event time data has become increasingly important in biomedical studies, especially in chronic diseases, HIV/AIDS, cardiovascular research, etc., when understanding the interplay between longitudinal biomarkers and clinical events is crucial. The joint modeling framework was developed by simultaneously modeling the longitudinal and event time data under a structure with shared subject‐specific random effects [11, 12, 13, 14, 15, 16, 17]. Recent development of the joint modeling method allows researchers to study the causal effect of the exposure on the survival outcome through the longitudinal marker [18, 19]. Previous work showed that by including both the random effect and the longitudinal marker in the survival submodel, the joint model framework allows the researcher to separate the direct causal effect of the longitudinal marker on the outcome and the time‐independent unmeasured confounding between the marker and the outcome [20, 21]. The maximum likelihood method equipped with the EM algorithm has been a popular approach to estimate parameters in a joint model [22] for making statistical inferences. Most joint modeling methods have been developed for longitudinal biomarkers and right‐censored event time, with an emphasis on either improving inference for event times through incorporation of time‐dependent longitudinal biomarkers [15, 16, 23] or estimating longitudinal biomarker trajectories in the presence of informative dropout [24, 25, 26]. These methods are not well‐suited for studying the relationship between cognitive impairments and HD onset in prodromal HD individuals, where the onset time is subject to interval censoring and is only known to occur between two consecutive motor assessments—the first yielding a negative diagnosis and the second confirming HD onset. Although joint models of longitudinal biomarkers and interval‐censored event times have been studied more recently [27, 28, 29, 30], the methods did not concern the changes of longitudinal biomarkers triggered by the event time. Some two‐phase change‐point analyses of longitudinal data around an interval‐censored event time have been explored [31, 32, 33], but they did not address how the longitudinal data impacted the interval‐censored event time. In addition, none of the aforementioned approaches were concerned with causal inferences.

In this work, we propose a two‐phase joint model for longitudinal biomarkers and interval‐censored event times that accommodates a change point anchored at an unobserved event time (see Figure 1) under the causal framework of Liu et al. [20]. The first phase of the model emphasizes how the longitudinal data impact the event time, and the second phase investigates whether/how the event time changes the trajectories of longitudinal biomarkers.

**FIGURE 1:**
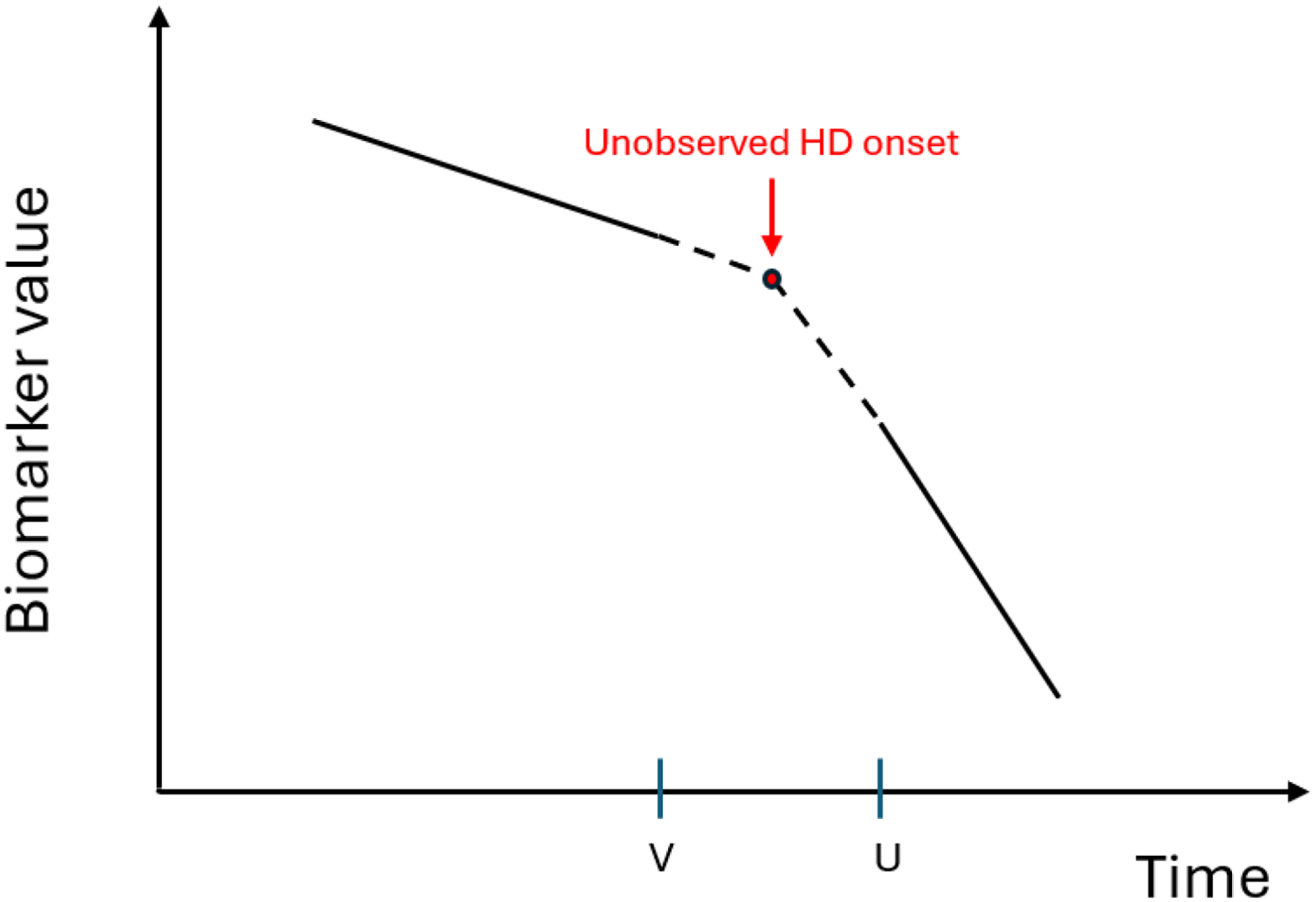
A hypothetical model for the HD disease progression: V and U are the two adjacent diagnosed times, with V being negative and U being positive for the HD diagnosis.

The rest of this paper is organized as follows: in Section 2, we describe our notation, models, and the likelihood method for the causal two‐phase joint modeling of change‐point longitudinal data and interval‐censored event time and illustrate how to calculate the MLE. In Section 3, we present simulation studies to evaluate the finite‐sample performance of the proposed method. In Section 4, we apply our method to PREDICT‐HD, a 12‐year multisite prospective cohort study conducted between September 2002 and April 2014 [3], to investigate the interplay between cognitive decline and HD onset in prodromal HD individuals, assessing whether cognitive declines predict the HD onset and whether HD onset subsequently accelerates cognitive declines. In Section 5, we summarize our findings and discuss potential extensions.

## 2 Methods

### 2.1 Notation

First, we define notation for observed data. We consider K‐dimensional longitudinal biomarker processes $M\left(t\right)=\left(M^{1}\left(t\right),M^{2}\left(t\right),\cdots ,M^{K}\left(t\right)\right)$ that are potentially predictive of the onset time of disease E, which is interval censored by (V,U] via periodic diagnoses, where V and U can be zero and infinity, respectively. Let X(t) be covariate processes that are potentially related to both longitudinal disease biomarkers and the onset time of disease. Assume the processes are assessed longitudinally at m random observation times $\underline{T}=\left(t_{1},t_{2},\cdots ,t_{m}\right)$, and the data from the processes collected at $\underline{T}$ are denoted as $\underline{M}=\left(M\left(t_{1}\right),M\left(t_{2}\right),\cdots ,M\left(t_{m}\right)\right)$ and $\underline{X}=\left(X\left(t_{1}\right),X\left(t_{2}\right),\cdots ,X\left(t_{m}\right)\right)$. The observed data for an individual consist of $D=\left(m,\underline{T};\underline{M},\underline{X};V,U\right)$, and we have a total of n independent and identically distributed (i.i.d.) copies of D, denoted by $\underline{D}=(D_{1},D_{2},\cdots ,D_{n}$). The observation times $\underline{T}$ and number of observations m may vary across individuals, accommodating irregular measurement schedules commonly encountered in real data applications.

### 2.2 Causal Two‐Phase Joint Model

In studying disease progression, we hypothesize that (i) the longitudinal disease biomarkers M(⋅) causally affect the onset time, E, and (ii) disease onset, in turn, may alter the subsequent trajectories of these biomarkers. To accommodate this potentially bidirectional relationship between the longitudinal biomarkers and disease onset, we propose a two‐phase framework for characterizing disease progression, as described below:

(1) $$M_{\infty }^{k}\left(t\right)=X{\left(t\right)}^{⊤}\beta^{k}+Z{\left(t\right)}^{⊤}a^{k}+\epsilon^{k}\left(t\right),\;$$

(2) $$\lambda \left(t\right)=\lambda_{0}\left(t\right)\exp\left[\theta_{x}^{⊤}X\left(t\right)+\theta_{a}^{⊤}a+\theta_{M}^{⊤}M_{\infty }\left(t\right)\right],$$

(3) $$M_{E}^{k}\left(t\right)=M_{\infty }^{k}\left(t\right)+I\left(t>E\right)g^{k}\left(t-E\right),\;$$

for k=1,2,⋯,K, where $M_{\infty }^{k}\left(\cdot \right)$ denotes the kth longitudinal biomarker process shall the disease never happen and $M_{E}^{k}\left(\cdot \right)$ denotes the kth post‐disease longitudinal biomarker process. By consistent assumption, the observed kth longitudinal biomarker process will satisfy $M^{k}\left(t\right)=M_{E}^{k}\left(t\right)$ after the disease has started at time E. Therefore we can divide M(t) into two phases in the likelihood calculation, where $M_{1}^{k}\left(t\right)=M_{\infty }^{k}\left(t\right),t\in \left(0,E\right)$ and $M_{2}^{k}\left(t\right)=M_{E}^{k}\left(t\right),t\in \left[E,\infty \right)$ denote the first (before E) and second (after E) phases of the kth longitudinal biomarker process, $M^{k}\left(\cdot \right)$, respectively.

In the two‐phase longitudinal submodel, $\beta^{k}$ represents the effects of the covariates on the kth biomarker; $a=\left(a^{1},a^{2},\cdots a^{K}\right)∼N\left(0,\Sigma_{a}\right)$ represent the random effects at the individual level, which are used to surrogate time‐independent unmeasured confounding between the longitudinal biomarkers and the survival outcome; Z(⋅) are the covariates of longitudinal processes corresponding to the random effects, which are often a part of X(⋅) in practice of the joint modeling; $\epsilon \left(t\right)=\left(\epsilon^{1}\left(t\right),\epsilon^{2}\left(t\right),\cdots ,\epsilon^{K}\left(t\right)\right)∼N\left(0,\Sigma_{e}\right)$ are i.i.d. time‐independent random variations for observed longitudinal data; and $g^{k}\left(\cdot \right)$ represents the change‐point effect anchored at the interval‐censored disease onset time, E, on the kth longitudinal biomarker.

For the survival submodel, $\lambda_{0}\left(t\right)$ represents an unspecified baseline hazard function for the onset time; $\theta_{x}$ represents the effects of the covariates on the onset time; $\theta_{a}$ represents the effects of the random effects on the survival outcome, which reflect whether unmeasured baseline variables confound the association between longitudinal biomarkers and survival outcome; and $\theta_{M}$ represents the effects of longitudinal biomarkers at the first phase on the onset time.

The proposed two‐phase joint model extends existing causal joint models for longitudinal and survival data [20, 21] by allowing disease onset to affect longitudinal biomarker trajectories and by accommodating interval‐censored disease‐onset times. In this paper, we focus on using the proposed framework to assess evidence supporting the hypothesized causal structure linking exposure, longitudinal biomarkers, and disease onset, rather than on quantifying causal effects through specific causal estimands, such as natural direct and indirect effects. Because the survival model incorporates $M_{\infty }^{k}\left(t\right)$ rather than $E\left(M_{\infty }^{k}\left(t\right)|a\right)$, the additional inclusion of a does not induce perfect collinearity, and the proposed model therefore remains identifiable under a specific parametric model for the shared random effect. However, these extensions result in a substantially more complex observed‐data likelihood and introduce considerable computational challenges that must be addressed in the methodological development.

### 2.3 The Likelihood for the Observed Data

In this subsection, we construct the likelihood function of the parameters $\Theta =\left(\lambda_{0}\left(\cdot \right),g\left(\cdot \right),\beta ,\theta_{X},\theta_{a},\theta_{M},\Sigma_{a},\Sigma_{e}\right)$ of the two‐phase joint models (1–3) for the observed data $\underline{D}$, where $\beta =\left(\beta^{1},\beta^{2},\cdots ,\beta^{K}\right)$ and $g\left(\cdot \right)=\left(g^{1}\left(\cdot \right),g^{2}\left(\cdot \right),\cdots ,g^{K}\left(\cdot \right)\right)$. As a conventional approach in joint modeling with shared random effects, we assume that given the shared random variables a, the observed data on the longitudinal biomarkers are independent within the same biomarker process and between different biomarker processes, as well as independent of the disease onset time.

We begin by deriving the likelihood for the case in which the disease onset time is observed exactly. The longitudinal biomarker profile, $\underline{M}$, is partitioned into the pre‐onset component, ${\underline{M}}_{1}=\left(M\left(t_{1}\right),M\left(t_{2}\right),\ldots ,M\left(V\right)\right)$, and the post‐onset component, ${\underline{M}}_{2}=\left(M\left(U\right),\ldots ,M\left(t_{m}\right)\right)$. Under the shared random‐effects models (1–3), the joint density admits the following factorization:

(4) $$\begin{array}{ll} & f\left(E,\underline{M}|m,\underline{T},\underline{X},a\right)\\ & \;=f\left({\underline{M}}_{2}|E,m,\underline{T},\underline{X},a\right)f\left(E|{\underline{M}}_{1},m,\underline{T},\underline{X},a\right)f\left({\underline{M}}_{1}|m,\underline{T},\underline{X},a\right), \end{array}$$

where

$$\begin{array}{ll} & f\left(E|{\underline{M}}_{1},m,\underline{T},\underline{X},a\right)=\lambda_{0}\left(E\right)\exp\left(\theta_{X}^{⊤}X\left(E\right)+\theta_{a}^{⊤}a+\theta_{M}^{⊤}M_{1}\left(E\right)\right)\\ & \;\times \exp\left(\left\{-\sum_{j:t_{j}\leq E}\int_{t_{j-1}}^{t_{j}}\lambda_{0}\left(s\right)\exp\left(\theta_{X}^{⊤}X\left(t_{j-1}\right)+\theta_{a}^{⊤}a+\theta_{M}^{⊤}M_{1}\left(t_{j-1}\right)\right)\mathrm{ds}\right\}\right), \end{array}$$

$$\begin{array}{ll} f\left({\underline{M}}_{1}|m,\underline{T},\underline{X},a\right) & =\prod_{j:t_{j}<E}\left[\frac{1}{\sqrt{{\left(2\pi \right)}^{K}\det\left(\Sigma_{e}\right)}}\right.\\ & \;\times \exp\left(-\frac{1}{2}{\left(M_{1}\left(t_{j}\right)-X{\left(t_{j}\right)}^{⊤}\beta -Z{\left(t_{j}\right)}^{⊤}a\right)}^{⊤}\Sigma_{e}^{-1}\right.\\ & \;\left.\left.\times \left(M_{1}\left(t_{j}\right)-X{\left(t_{j}\right)}^{⊤}\beta -Z{\left(t_{j}\right)}^{⊤}a\right)\right)\right], \end{array}$$

and

$$\begin{array}{ll} & f\left({\underline{M}}_{2}|E,m,\underline{T},\underline{X},a\right)=\prod_{j:t_{j}\geq E}\left[\frac{1}{\sqrt{{\left(2\pi \right)}^{K}\det\left(\Sigma_{e}\right)}}\right.\\ & \;\times \exp\left(-\frac{1}{2}{\left(M_{2}\left(t_{j}\right)-X{\left(t_{j}\right)}^{⊤}\beta -Z{\left(t_{j}\right)}^{⊤}a-g\left(t_{j}-E\right)\right)}^{⊤}\Sigma_{e}^{-1}\right.\\ & \;\times \left.\left.\left(M_{2}\left(t_{j}\right)-X{\left(t_{j}\right)}^{⊤}\beta -Z{\left(t_{j}\right)}^{⊤}a-g\left(t_{j}-E\right)\right)\right)\right]. \end{array}$$

We note that evaluation of $f\left(E,\underline{M}|m,\underline{T},\underline{X},a\right)$ requires the complete trajectories of X(⋅) and $M_{1}\left(\cdot \right)$, whereas these processes are observed only at the discrete time points $\underline{T}$. Accordingly, we employ a last‐observation‐carried‐forward approximation, replacing their values between consecutive observation times $t_{j-1}$ and $t_{j}$ by their values at $t_{j-1}$ in the above expression of $f\left(E,\underline{M}|m,\underline{T},\underline{X},a\right)$.

Since the disease onset time E is interval‐censored by (V,U] in our study of the disease progression for prodromal HD individuals, with U possibly equal to infinity for individuals who remain undiagnosed at the end of follow‐up, we introduce a binary indicator Δ, where Δ=1 if E∈(V,U], with V and U corresponding to two consecutive observation times, and Δ=0 if E is right‐censored at the end of follow‐up, in which case $V=t_{m}$ and U=∞. Hence,

$$\begin{array}{ll} & f\left(\underline{M},V<E\leq U|m,\underline{T},\underline{X},a\right)\\ & \;={\left[\int_{V}^{U}f\left({\underline{M}}_{2}|t,m,\underline{T},\underline{X},a\right)f\left(t|{\underline{M}}_{1},m,\underline{T},\underline{X},a\right)\mathrm{dt}\right]}^{\Delta }\\ & \;\times S{\left(V|{\underline{M}}_{1},m,\underline{T},\underline{X},a\right)}^{1-\Delta }f\left({\underline{M}}_{1}|m,\underline{T},\underline{X},a\right), \end{array}$$

where

$$\begin{array}{ll} & S\left(V|{\underline{M}}_{1},m,\underline{T},X,a\right)\\ & \;=\exp\left\{-\sum_{j:t_{j}\leq V}\int_{t_{j-1}}^{t_{j}}\lambda_{0}\left(s\right)\exp\left(\theta_{x}^{⊤}X\left(t_{j-1}\right)+\theta_{a}^{⊤}a+\theta_{m}^{⊤}M_{1}\left(t_{j-1}\right)\right)\mathrm{ds}\right\}. \end{array}$$

Assuming that the number of observations, observation times, and the covariate processes are noninformative to the model parameters Θ, the likelihood for D as a function of Θ is

$$\begin{array}{ll} L\left(\Theta ;D\right) & =\int {\left[\int_{V}^{U}f\left({\underline{M}}_{2}|t,m,\underline{T},\underline{X},a\right)f\left(t|{\underline{M}}_{1},m,\underline{T},\underline{X},a\right)\mathrm{dt}\right]}^{\Delta }\\ & \;\times S{\left(V|{\underline{M}}_{1},m,\underline{T},\underline{X},a\right)}^{1-\Delta }f\left({\underline{M}}_{1}|m,\underline{T},\underline{X},a\right)f\left(a;\Sigma_{a}\right)\mathrm{da}, \end{array}$$

where

$$f\left(a;\Sigma_{a}\right)={\left(2\pi \right)}^{-K/2}{\left(\det\left(\Sigma_{a}\right)\right)}^{-1/2}\exp\left(-\frac{1}{2}a^{⊤}\Sigma_{a}^{-1}a\right),$$

and the likelihood for the observed data $\underline{D}$ is

(5) $$\begin{array}{ll} L\left(\Theta ;\underline{D}\right) & =\prod_{i=1}^{n}L\left(\Theta ;D_{i}\right)\\ & =\prod_{i=1}^{n}\int {\left[\int_{V_{i}}^{U_{i}}f\left({\underline{M}}_{i2}|t,m_{i},{\underline{T}}_{i},{\underline{X}}_{i},a_{i}\right)f\left(t|{\underline{M}}_{i1},m_{i},{\underline{T}}_{i},{\underline{X}}_{i},a_{i}\right)\mathrm{dt}\right]}^{\Delta_{i}}\\ & \;\times S{\left(V_{i}|{\underline{M}}_{i1},m_{i},{\underline{T}}_{i},{\underline{X}}_{i},a_{i}\right)}^{1-\Delta_{i}}f\left({\underline{M}}_{i1}|m_{i},{\underline{T}}_{i},{\underline{X}}_{i},a_{i}\right)f\left(a_{i};\Sigma_{a}\right){\mathrm{da}}_{i}. \end{array}$$

We implemented a spline‐based semiparametric sieve maximum likelihood method to estimate the model parameters Θ by approximating the baseline cumulative hazard function $\Lambda_{0}\left(t\right)=\int_{0}^{t}\lambda_{0}\left(u\right)\mathrm{du}$ using the cubic monotone B‐spline

$$\Lambda_{0}\left(t\right)=\sum_{j=1}^{q_{n}}\alpha_{j}B_{j}\left(t\right),\;0\leq \alpha_{1}\leq \alpha_{2}\leq \cdots \leq \alpha_{q_{n}}.$$

where ${\left\{\alpha_{j}\right\}}_{j=1}^{q_{n}}$ are the coefficients of the B‐spline that are non‐negative and monotone non‐decreasing, and $q_{n}$ is the number of basis functions that depends on the sample size. The monotonicity of the B‐spline coefficients warrants the monotone non‐decreasing property of $\Lambda_{0}\left(\cdot \right)$ [34]. This spline‐based semiparametric sieve maximum likelihood estimation method has been widely employed for many semiparametric models [35, 36, 37, 38].

### 2.4 Numerical Algorithm

For the spline‐based semiparametric sieve maximum likelihood estimation, the model parameters become

$$\Theta =\left(\alpha ,\beta ,\gamma ,\theta_{x},\theta_{a},\theta_{M},\Sigma_{a},\Sigma_{e}\right),$$

with $\alpha =\left(\alpha_{1},\alpha_{2},\cdots ,\alpha_{q_{n}}\right)$ being non‐negative and monotone non‐decreasing and γ being the vector of unknown parameters associated with the change‐point effects of g(⋅). We assumed there are a total of r parameters in the model and adopted a general Fisher scoring algorithm with a line search procedure to compute the estimates:

$$\Theta^{\left(p+1\right)}=\Theta^{\left(p\right)}-\eta I^{-1}\left(\Theta^{\left(p\right)}\right)\dot{l}\left(\Theta^{\left(p\right)}\right),$$

where $\dot{l}\left(\Theta^{\left(p\right)}\right)=\nabla_{\Theta }l\left(\Theta^{\left(p\right)};\underline{D}\right)=\sum_{i=1}^{n}\nabla_{\Theta }l\left(\Theta^{\left(p\right)};D_{i}\right)=\sum_{i=1}^{n}\nabla_{\Theta }\log L\left(\Theta^{\left(p\right)};{\underline{D}}_{i}\right)$ represents the score of the log‐likelihood function, and $I\left(\Theta^{\left(p\right)}\right)=\sum_{i=1}^{n}\dot{l}\left(\Theta^{\left(p\right)};D_{i}\right)\dot{l}{\left(\Theta^{\left(p\right)};D_{i}\right)}^{T}$ represents the observed information matrix, where $\dot{l}\left(\Theta^{\left(p\right)};D_{i}\right)=\nabla_{\Theta }l\left(\Theta^{\left(p\right)};D_{i}\right)=\nabla_{\Theta }\log L\left(\Theta^{\left(p\right)};D_{i}\right)$. η is an adaptive step length when updating the parameters. We used the step‐halving adaptive line search strategy to ensure that the log‐likelihood increases after each iteration. We stopped the algorithm when the maximum relative difference of the consecutive updated parameter estimates was less than $10^{-3}$, that is, $\max_{\Theta }|\Theta^{\left(p+1\right)}-\Theta^{\left(p\right)}|/|\Theta^{\left(p\right)}|<10^{-3}$.

Although the proposed algorithm is conceptually straightforward, its numerical implementation entails several challenges. We addressed these challenges using the following practical strategies. First, it was inconvenient to perform the Fisher scoring algorithm with monotone constraints on ${\left\{\alpha_{j}\right\}}_{j=1}^{q_{n}}$. We used a reparameterization approach to remove the constraints in optimization by letting $\alpha_{j}=\sum_{l=1}^{j}\exp\left(\xi_{l}\right)$, for $j=1,2,\cdots ,q_{n}$. Second, there was no closed form for the integration over the interval $\left(V_{i},U_{i}\right)$ since the function to be integrated includes a product of exponentials of cubic B‐splines and a polynomial function of time t. To calculate this integral as accurately as possible, we choose to use Gauss‐Legendre quadrature, which has been proven to be reliable for approximating the bounded integral of a smooth function. We used 20 nodes inside $\left(V_{i},U_{i}\right)$ for the Gauss‐Legendre quadrature computation. There is also no closed‐form of the integration over random effects a. We used a 20‐node Gauss–Hermite quadrature, which is well‐suited for evaluating integrals of forms of $e^{-x^{2}}f\left(x\right)$. Third, it is a daunting job to calculate the analytical score function and Hessian matrix for the likelihood (5). We used the finite difference method to approximate the score:

$${\dot{l}}_{u}^{*}\left(\Theta^{\left(p\right)};D_{i}\right)=\frac{l\left(\Theta^{\left(p\right)}+\delta \cdot 1_{u};D_{i}\right)-l\left(\Theta^{\left(p\right)}-\delta \cdot 1_{u};D_{i}\right)}{2\delta },$$

where $1_{u}$ is a vector with 1 for the uth element and 0 for the other elements (1≤u≤r), and ${\dot{l}}_{u}^{*}$ is the finite difference of the log likelihood with δ chosen to be a very small value in the magnitude of $10^{-6}$ in our calculation. Consequently, the observed Fisher information matrix was approximated by $I^{*}\left(\Theta^{\left(p\right)}\right)=\sum_{i=1}^{n}{\dot{l}}^{*}\left(\Theta^{\left(p\right)};D_{i}\right){\dot{l}}^{*}{\left(\Theta^{\left(p\right)};D_{i}\right)}^{⊤}$, where ${\dot{l}}^{*}\left(\Theta^{\left(p\right)};D_{i}\right)={\left({\dot{l}}_{1}^{*}\left(\Theta^{\left(p\right)};D_{i}\right),\cdots ,{\dot{l}}_{r}^{*}\left(\Theta^{\left(p\right)};D_{i}\right)\right)}^{⊤}$. Last, the eigenvalues of the observed information matrix $I^{*}\left(\Theta^{\left(p\right)}\right)$ could be very small during the numerical iteration, causing potential troubles in inverting the approximated Fisher information matrix $I^{*}\left(\Theta^{\left(p\right)}\right)$. We used the generalized inverse matrix of ${\left(I^{*}\right)}^{-}\left(\Theta^{\left(p\right)}\right)$ instead, for the Fisher scoring algorithm.

For the initial values of Θ, we fitted longitudinal and survival submodels separately. First, we fitted a longitudinal model for each biomarker with fixed and random effects and the change‐point effect at E=(U+V)/2. Then, we fitted an ordinary Cox model for right‐censored data with E=(U+V)/2 as the known event time, and included both fixed covariates and estimated random effects from the longitudinal model as predictors. We used the JM packages [22] to combine the longitudinal and survival models to get an initial joint estimate of Θ except the baseline hazard, using the estimated parameters obtained above. To obtain the initial B‐spline estimate for the cumulative hazard function, we pre‐specified the number of knots $q_{n}$ in the order of $q^{1/3}$ with q being the total number of distinct values in the set of $\left\{U_{i},V_{i}|i=1,\ldots ,n\right\}$ [33], we used the corresponding quantiles, $t_{1},t_{2},\ldots ,t_{q_{n}-4}$ of the distinct values in the set of $\left\{U_{i},V_{i}|i=1,\ldots ,n\right\}$, as interior knots and applied the Maximum Likelihood Estimation (MLE) method for interval‐censored survival data and *the optim* function in R to complete the setting of initial values of $\Theta ,\Theta^{\left(0\right)}$. We used the nonparametric Bootstrap method to estimate the standard errors of the estimated Θ. The complete computing algorithm is summarized in the following flowchart (Algorithm 1).

ALGORITHM 1 **Sieve MLE for the two‐phase joint model.**

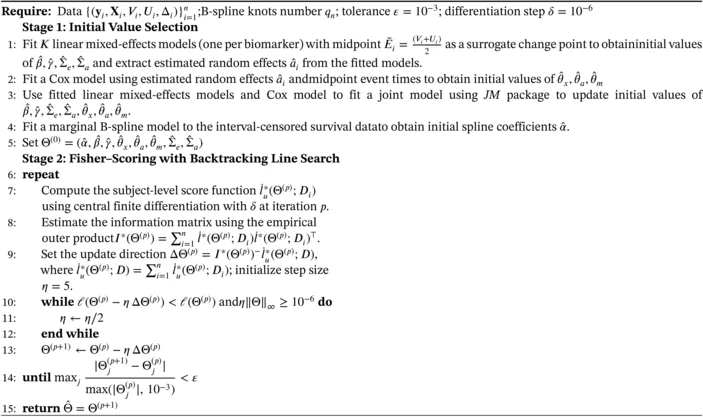

## 3 Simulation

### 3.1 Settings

We conducted a comprehensive simulation study to evaluate the performance of our proposed two‐phase model. For the ith individual, the observed data were generated from the following specific model of the two‐phase joint model (1–3): 


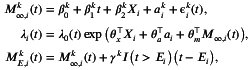


where we considered two biomarkers (k = 1,2) for the longitudinal model. A single time‐independent covariate was simulated according to $X_{i}∼N\left(0,0.8\right)$, and two random effects $a_{i}=\left(a_{i}^{1},a_{i}^{2}\right)$ were generated from a joint normal distribution $a_{i}∼N\left(0,\Sigma_{a}\right)$.

For the simulation study, we set $\beta_{0}={\left(5,5\right)}^{⊤},\beta_{1}={\left(-0.2,-0.2\right)}^{⊤},\beta_{2}={\left(-0.3,-0.3\right)}^{⊤},\gamma ={\left(-0.4,-0.4\right)}^{⊤},\theta_{x}=0.3,$ $\theta_{a}={\left(0.1,0.1\right)}^{⊤},\theta_{m}={\left(-0.2,-0.2\right)}^{⊤},\Sigma_{a}=\left(\begin{array}{cc} 0.5 & 0.1\\ 0.1 & 0.5 \end{array}\right),\Sigma_{e}=\left(\begin{array}{cc} 0.5 & \frac{\sqrt{2}}{10}\\ \frac{\sqrt{2}}{10} & 1 \end{array}\right),$ and $\lambda_{0}\left(t\right)=0.3\sqrt{t/5}$ to generate the data. m=11 observations were simulated for each individual, with the observation times ${\underline{T}}_{i}={\left\{t_{\mathrm{ij}}\right\}}_{j=1}^{10}$ independently generated from uniform distributions U(j−0.2,j+0.2),j=1,2,…,10, respectively.

For the simulation study, we modeled the change‐point effects as $g^{k}\left(t-E\right)=\gamma^{k}\left(t-E\right)$, allowing the longitudinal biomarker trajectories to exhibit changes in slope after the event time E, which reflects our objective for the motivating PREDICT‐HD study.

In the simulation study, we used cubic B‐splines with 6 and 10 interior knots to carry out the spline‐based semiparametric maximum sieve likelihood method to evaluate the sensitivity of the analysis results to the choice of the number of spline knots. We conducted 1000 simulation replicates for sample sizes of 400 and 800 to evaluate the finite‐sample performance of the proposed method and assess the adequacy of the asymptotic normal approximation. The results are summarized in terms of the empirical bias, Monte Carlo standard deviation (SD), average bootstrap standard error estimate (ASE) based on 50 bootstrap samples, and coverage probability (CP) of the 95% Wald confidence interval. The simulation study was conducted using the Holland Computer Center (HCC) server at the University of Nebraska, Lincoln. All codes were written in the R module 4.4.

### 3.2 Results

Table 1 displays the results of the spline‐based semiparametric maximum sieve likelihood analysis for the simulated data, where the cumulative hazard function was approximated by a cubic B‐spline with six interior knots. It appears that the estimation biases of the regression coefficients were negligible; the average bootstrap standard error estimates were all very close to the corresponding Monte‐Carlo empirical standard deviations, indicating that the bootstrap estimation of standard error was reliable; and the 95% Wald confidence intervals yielded a right coverage probability of 0.95. As the sample size increased from 400 to 800, both the empirical standard deviations and the average bootstrap standard error estimates decreased by approximately $1/\sqrt{2}$, as expected under the asymptotic normality theory. These results support the validity of asymptotic normal inference for the regression coefficient estimators in the proposed two‐phase joint model.

**TABLE 1:** Simulation results for the estimated regression coefficients from the two‐phase joint model using the spline‐based semiparametric maximum sieve likelihood method with six interior knots.

*n* = 400	Parameters	Bias	SD	ASE	CP
Biomarker 1	$\beta_{0}$	−0.002	0.042	0.044	0.956
	$\beta_{1}$	0.000	0.004	0.004	0.949
	$\beta_{2}$	0.000	0.043	0.045	0.935
	γ	0.000	0.011	0.011	0.947
Biomarker 2	$\beta_{0}$	0.002	0.045	0.046	0.949
	$\beta_{1}$	−0.000	0.006	0.006	0.937
	$\beta_{2}$	−0.002	0.044	0.043	0.949
	γ	0.000	0.015	0.015	0.940
Survival model	$\theta_{x}$	0.011	0.090	0.094	0.954
	$\theta_{m1}$	0.005	0.112	0.114	0.950
	$\theta_{m2}$	0.001	0.082	0.080	0.940
	$\theta_{a1}$	−0.005	0.167	0.175	0.955
	$\theta_{a2}$	0.002	0.154	0.161	0.952

*n* = 800	Parameters	Bias	SD	ASE	CP
Biomarker 1	$\beta_{0}$	0.001	0.031	0.031	0.943
	$\beta_{1}$	0.000	0.003	0.003	0.944
	$\beta_{2}$	−0.001	0.040	0.040	0.941
	γ	0.000	0.009	0.009	0.936
Biomarker 2	$\beta_{0}$	−0.000	0.039	0.040	0.940
	$\beta_{1}$	0.000	0.006	0.006	0.951
	$\beta_{2}$	0.001	0.029	0.031	0.953
	γ	0.000	0.008	0.008	0.939
Survival model	$\theta_{x}$	0.006	0.069	0.066	0.924
	$\theta_{m1}$	0.002	0.082	0.080	0.939
	$\theta_{m2}$	−0.001	0.059	0.057	0.936
	$\theta_{a1}$	0.003	0.128	0.122	0.936
	$\theta_{a2}$	−0.003	0.111	0.112	0.951

Figures 2 and 3 display the estimated cumulative hazard function obtained using the B‐splines with six interior knots for sample sizes of n=400 and 800, respectively. The red dashed curve represents the true cumulative hazard function, while the blue solid curve denotes the pointwise average of the estimated cumulative hazard function across the 1000 replicates. The light‐blue band represents the pointwise 95% simulation envelope, with the lower and upper limits corresponding to the 2.5% and 97.5% percentiles, respectively, of the pointwise estimates across the 1000 replicates. It is evident that the estimated cumulative hazard function closely aligned with the true function, demonstrating the consistency of the proposed B‐spline estimator. Moreover, the simulation envelope became noticeably narrower as the sample size increased, indicating improved estimation precision.

**FIGURE 2:**
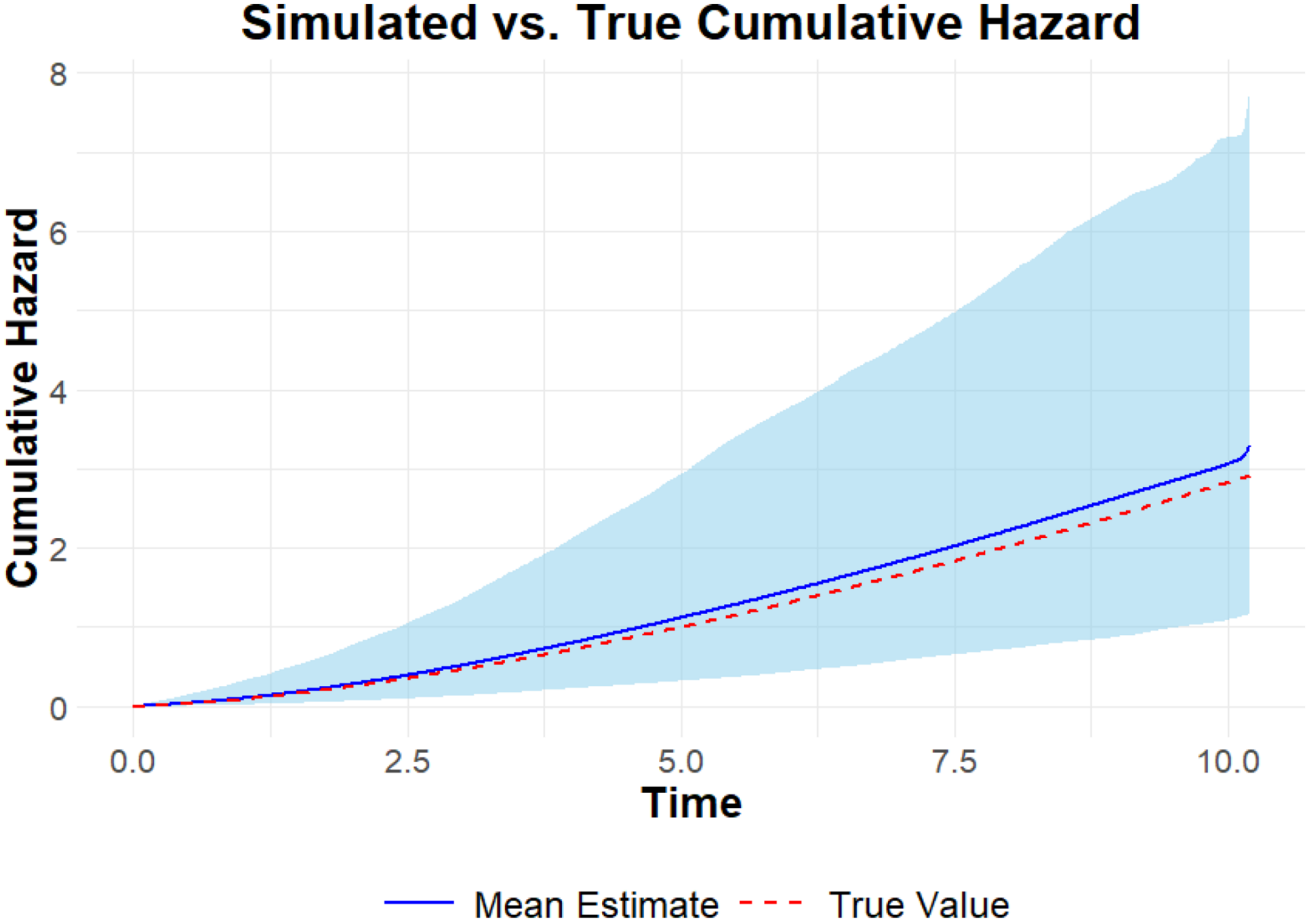
The summary of the 1000 estimated cubic B‐spline cumulative hazard functions with six interior knots: *N* = 400, true $\Lambda_{0}\left(t\right)={\left(0.2t\right)}^{1.5}$.

**FIGURE 3:**
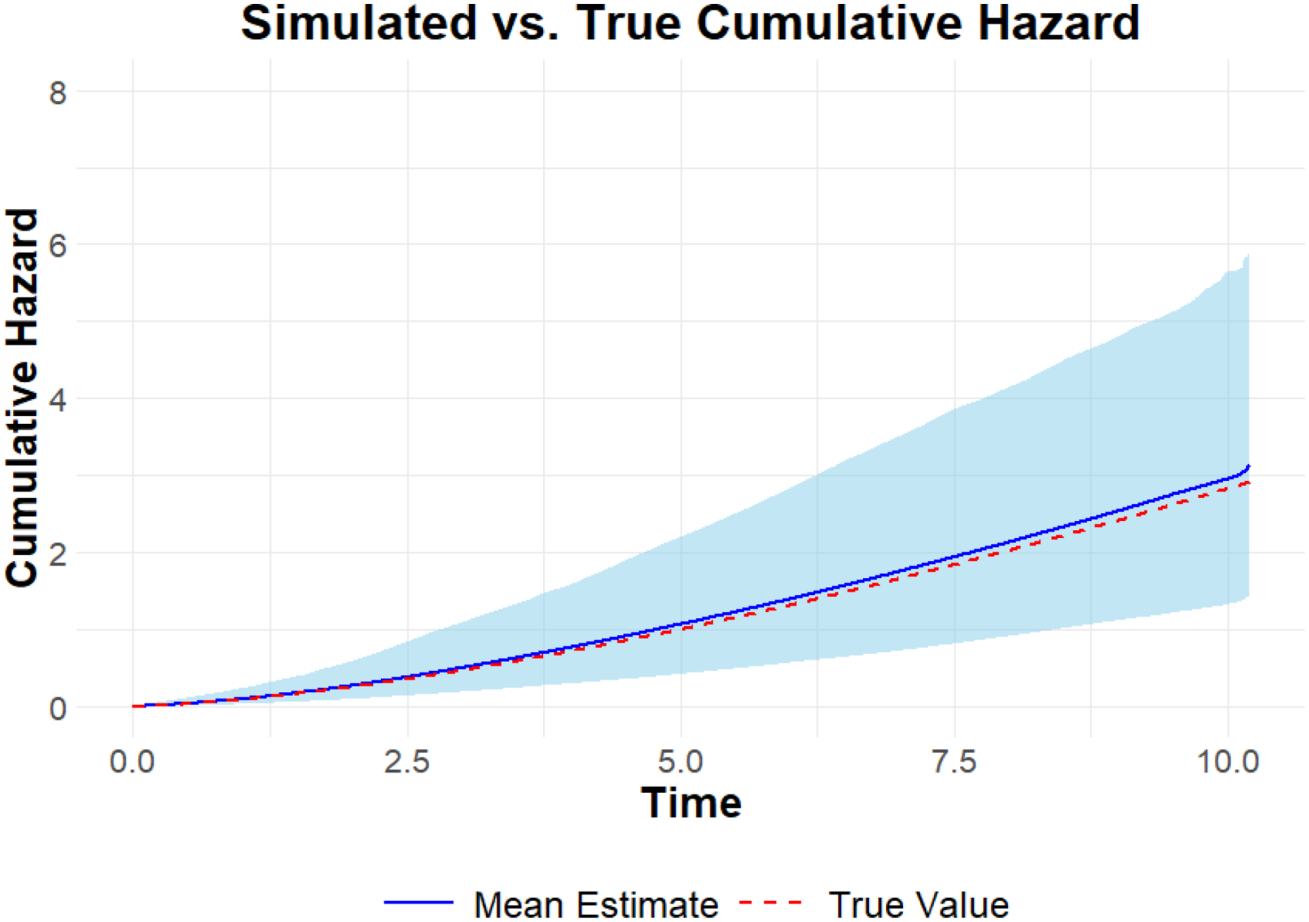
The summary of the 1000 estimated cubic B‐spline cumulative hazard functions with six interior knots: *N* = 800, true $\Lambda_{0}\left(t\right)={\left(0.2t\right)}^{1.5}$.

Additional simulation results (not shown here) with 10 interior knots in the cubic B‐spline approximation for the baseline cumulative hazard function $\Lambda_{0}\left(\cdot \right)$ were similar to those shown in Table 1 and Figures 2 and 3. These findings suggest that the performance of the proposed spline‐based semiparametric maximum sieve likelihood method is insensitive to the choice of the number of spline knots when $\Lambda_{0}\left(\cdot \right)$ is smooth.

## 4 A Real‐World Application: Predict‐HD Study

### 4.1 Data and Study Objectives

We applied our proposed causal two‐phase joint model to data from the Neurobiological Predictors of Huntington's Disease (PREDICT‐HD) study, a 12‐year multisite prospective cohort study conducted from September 2002 to April 2014 [3]. The study recruited 1155 individuals with the HD gene expansion and 317 controls without the HD gene expansion, mainly family members of the recruited HD gene–expanded individuals. The primary objective of this analysis was to evaluate two central hypotheses: (1) that cognitive decline predicts the time to motor onset during the prodromal stage of HD, and (2) that the motor onset serves as a change point that accelerates the subsequent rate of cognitive decline.

In the PREDICT‐HD study, the clinical diagnosis of HD was determined by a certified motor assessor trained by the Huntington Study Group (HSG), who evaluated each participant using the Diagnostic Confidence Level (DCL) [39]. A DCL score of four indicates greater than 99% confidence that the observed motor abnormalities are unequivocal signs of HD and is therefore considered a positive diagnosis of HD motor onset, whereas a DCL score less than four is considered a negative diagnosis. The PREDICT‐HD data are particularly well suited to the proposed model because the DCL was only assessed at scheduled clinical visits, and the time to HD onset was known only to lie within the interval between two consecutive visits. Consequently, the HD onset time is naturally interval‐censored by an interval (V,U] for the study participants of prodromal HD individuals.

We evaluated two key longitudinal cognitive biomarkers in studying HD progression [9]: the Symbol Digit Raw Score Total (*sydigtot*), which measures processing speed and attention, and the Stroop Word Reading Total (*stroopwo*), which assesses information integration and inhibition. Four baseline covariates were included in the analysis: age at study entry, years of education, gender (0 = Female, 1 = Male), and the HD disease burden (CAP) score. The CAP score, which combines age and CAG repeat length, is a well‐established prognostic measure [8]. For more meaningful interpretability in analysis results, the CAP score was rescaled by dividing it by 100.

After excluding individuals who were diagnosed at their baseline visit or had only a single longitudinal assessment, the final analysis dataset comprised 983 prodromal HD individuals, contributing a total of 5694 longitudinal observations.

### 4.2 Results

We fit the proposed causal two‐phase joint model using a cubic B‐spline with six interior knots to approximate the baseline cumulative hazard function. 100 bootstrap samples were used to estimate the standard errors and calculate *p* values. The analysis results are presented in Table 2 for the longitudinal submodel and Table 3 for the survival submodel. Overall, this analysis provides strong empirical support for the hypothesized relationships between cognitive decline and HD motor onset for prodromal HD individuals.

**TABLE 2:** Analysis results of the longitudinal submodel for the PREDICT‐HD data using the spline‐based semiparametric maximum sieve likelihood method for the proposed two‐phase model: A cubic B‐spline with six interior knots was used to approximate the baseline cumulative hazard function, and the standard errors of the estimated regression coefficients were estimated using the nonparametric bootstrap method with 100 resamples.

			Bootstrap 95% CI		
	Coefficients	Estimate	Lower	Upper	*p*
Longitudinal model for sydigtot	Intercept	59.366	51.946	66.785	0.000
	Time	−0.553	−0.629	−0.476	0.000
	Age	−0.116	−0.193	−0.038	0.004
	Education	1.210	0.864	1.557	0.000
	Gender	−1.553	−3.520	0.146	0.073
	CAP/100	−5.835	−7.115	−4.556	0.000
	Time after diagnosis	−1.307	−1.620	−0.994	0.000
Longitudinal model for stroopwo	Intercept	101.930	90.165	113.695	0.000
	Time	−0.856	−0.991	−0.721	0.000
	Age	0.086	−0.056	0.228	0.235
	Education	1.607	0.941	2.272	0.000
	Gender	−2.265	−4.752	0.221	0.074
	CAP/100	−8.462	−10.137	−6.787	0.000
	Time after diagnosis	−2.067	−2.663	−1.472	0.000

Abbreviation: CI, confidence interval.

**TABLE 3:** Analysis results of the survival submodel for the PREDICT‐HD data using the spline‐based semiparametric maximum sieve likelihood method for the proposed two‐phase model: A cubic B‐spline with six interior knots was used to approximate the baseline cumulative hazard function, and the standard errors of the estimated regression coefficients were estimated using the nonparametric bootstrap method with 100 resamples.

			Bootstrap 95% CI		
	Coefficients	HR	Lower	Upper	*p*
Survival model	Age	0.998	0.982	1.014	0.784
	Education	1.005	0.941	1.073	0.887
	Gender	0.737	0.561	0.968	0.028
	CAP/100	2.232	1.594	3.125	0.000
	Sydigtot	0.954	0.916	0.992	0.019
	Stroopwo	0.988	0.970	1.006	0.184
	Random sydigtot	1.004	0.955	1.055	0.882
	Random stroopwo	0.997	0.971	1.025	0.854

Abbreviation: HR, hazard ratio.

The survival submodel (Table 3) examined the association between early cognitive decline and time to HD motor onset. The results support our first hypothesis. After adjusting for CAP score, *sydigtot* was a significant predictor of HD motor onset, with a hazard ratio (HR) of 0.954 (95% CI: 0.916, 0.992, p=0.019). In contrast, *stroopwo* was a weaker predictor, with an HR of 0.988 (95% CI: 0.970, 1.006, p=0.184). Hazard ratios less than 1.0 indicate that better cognitive performance (higher cognitive scores) is associated with a lower instantaneous risk of HD motor onset, whereas poorer cognitive performance is associated with a higher hazard and an earlier onset of HD symptoms.

As expected, the baseline CAP score was the primary driver of HD motor onset (HR = 2.232, 95% CI: 1.594, 3.125, p<0.0001), confirming that greater baseline disease burden strongly predicted HD motor onset. We also observed a significant effect of gender (HR = 0.737, p=0.028), suggesting that males had a lower hazard of HD motor onset in this cohort.

The longitudinal submodel (Table 2) characterized the two‐phase progression of cognitive biomarkers, with the unobserved HD motor onset time E serving as the change point. The results provide strong support for our second hypothesis. The change‐point effect (γ) was highly significant and negative for both *sydigtot* ($\hat{\gamma }=-1.307$, 95% CI: −1.620, −0.994, p<.0001) and *stroopwo* ($\hat{\gamma }=-2.067$, 95% CI: −2.663, −1.472, p<.0001),indicating that cognitive decline accelerated significantly following HD motor onset. For example, the *stroopwo* score declined at a baseline rate of 0.856 units per year before the motor onset. Following the motor onset, the rate of decline increased by an additional 2.067 units per year, yielding an overall post‐onset rate of decline of 2.923 units per year. These findings indicate that HD motor onset is not merely a clinical milestone but also marks a substantial acceleration in cognitive decline. Together, these results provide strong empirical support for the two‐phase progression illustrated in Figure 1.

The estimated covariate effects in the longitudinal submodel were also clinically consistent. A higher baseline CAP score (greater disease burden) and older age were both associated with significantly lower cognitive scores. In contrast, higher education attainment was associated with significantly higher cognitive scores, consistent with the well‐established association between education and better performance in neuropsychologic tests.

Another notable finding from the survival submodel (Table 3) was the non‐significance of the shared random effects (random *sydigtot*, p=0.882; random *stroopwo*, p=0.854). In the joint modeling framework [21], these shared random effects represent unmeasured time‐independent confounding between the longitudinal and survival processes that is not explained by the observed cognitive trajectories. Their non‐significance suggests that, after accounting for the observed cognitive trajectory, $M_{1}\left(t\right)$, the underlying shared variability (i.e., the random intercepts $a^{k}$) does not provide additional information for predicting HD motor onset. This finding supports the interpretation that the association between cognitive decline and HD motor onset is primarily explained by the observed cognitive trajectories, rather than by an unmeasured confounder influencing both cognition and motor onset.

The primary analysis used a central finite‐difference step size of $\delta =10^{-6}$ for numerical differentiation and 20‐node Gauss–Hermite quadratures for numerical integration associated with the unobserved random effects $a_{i}$. The analysis results may depend on the tuning parameters associated with these numerical approximations. Therefore, we conducted sensitivity analyses to evaluate its robustness to the choice of these tuning parameters. In the sensitivity analyses, we additionally considered step sizes of $\delta =10^{-4}$ and $10^{-5}$ and quadrature rules with 15 and 25 nodes. Changing the finite‐difference step size resulted in relative differences of less than 0.1% for all estimated regression coefficients, indicating that our algorithm is robust to different step sizes. The algorithm appeared to be somewhat sensitive to the choice of the number of Gauss–Hermite nodes. In the longitudinal submodel, some coefficients such as Age, Gender, and CAP score had a relatively large change (> 25%). However, the signs of these estimated coefficients remained unchanged. Additionally, the estimated time effects both before and after HD motor onset in the longitudinal submodel, as well as all estimated regression coefficients in the HD motor‐onset submodel, changed little. Thus, the sensitivity to the choice of the number of Gauss–Hermite nodes would not materially alter the substantive inferences drawn from the PREDICT‐HD study regarding disease progression.

In addition, we assessed the sensitivity of the estimation procedure to the choice of initial values used in the Fisher scoring algorithm. Specifically, we perturbed the initial parameter values selected according to the proposed algorithm by setting

$$\Theta_{\text{sens}}^{\left(0\right)}=\Theta^{\left(0\right)}+r\cdot \Theta^{\left(0\right)},$$

where r∼U(−R,R) with R = 0.25 and 0.5, respectively. These perturbed values were then used as the starting values for the Fisher scoring algorithm. Across all settings, the relative differences in all the estimated regression parameters were less than 0.1%, indicating the estimation procedure was highly robust to the choice of initial values.

In summary, application of the proposed two‐phase joint model to the PREDICT‐HD study elucidated the bidirectional relationship between cognitive decline and HD motor onset. The results strongly support the two hypothesized causal mechanisms in the HD disease progression for prodromal HD individuals: cognitive decline is an important early predictor of HD motor onset and that HD motor onset, in turn, serves as a change point that is associated with a marked acceleration in subsequent cognitive decline.

## 5 Final Remarks

Using the spline‐based sieve likelihood approach, the proposed causal two‐phase joint model yielded reliable inference for the regression coefficients, as demonstrated by the simulation study. Specifically, the spline‐based semiparametric maximum sieve likelihood estimators exhibited satisfactory consistency; the 95% confidence intervals for the regression coefficients achieved coverage probabilities close to the nominal level; the empirical standard deviations decreased at approximately the expected $n^{-1/2}$ rate, consistent with asymptotic normality; and the bootstrap standard errors closely agreed with the corresponding empirical standard deviations. The simulation results provide empirical support for the asymptotic normal approximation of the estimated regression coefficients in the proposed model. Although a rigorous theoretical justification has not yet been established, the simulation findings suggest that the asymptotic theory provides an accurate approximation in finite samples. Establishing the corresponding asymptotic theory remains an important direction for future research.

Applying our proposed method to the PREDICT‐HD data, we successfully characterized disease progression in individuals with prodromal HD. Our analysis showed that both cognitive functions—processing speed/attention (*sydigtot*) and information integration/inhibition (*stroopwo*)—declined over time, and that *sydigtot* was a significant predictor of HD motor onset. Moreover, the rate of decline in both cognitive domains accelerated after motor onset. These findings provide important insights into the natural history of HD and demonstrate the utility of the proposed method for characterizing the dynamic relationship between cognitive decline and HD motor onset.

While the proposed method was illustrated using a linear time effect to facilitate interpretation of the change‐point effect in the PREDICT‐HD application, it can be readily extended to accommodate nonlinear time effects with change‐point effects $g^{k}\left(\cdot \right)$ for k=1,⋯,K chosen to be any predetermined smooth functions. This extension does not fundamentally alter the numerical implementation, because the integrand between interval $\left(V,U\right):f\left(M_{2}|t,X,Z,a;\beta ,\gamma ,\Sigma_{e}\right)f\left(t|M_{1},X,Z,a;\theta ,\lambda \right)$ remains a smooth function of time t, and the Gauss‐Legendre quadrature continues to provide an accurate and effective method to approximate the likelihood integral over the interval (V,U). In addition, the proposed framework can be further extended by approximating both the baseline longitudinal trajectories and the change‐point effects, $g^{k}\left(\cdot \right)$ for k=1,2,⋯,K, using cubic B‐splines. This leads to a fully semiparametric sieve maximum likelihood approach that flexibly captures nonlinear biomarker trajectories without imposing parametric functional forms, making it particularly useful when accurately characterizing the underlying disease progression is of primary scientific interest.

Our proposed method did not explicitly account for measurement errors in the longitudinal biomarkers. In many practical problems, however, longitudinal biomarkers are often measured with error. Consequently, the observed biomarker process used in the survival component may not accurately represent the underlying biomarker trajectories, and the residual variation in the longitudinal model reflects both true within‐subject variability and measurement error. A natural extension is to incorporate a measurement error model and replace the observed biomarker process, $M_{\infty }^{k}\left(t\right)$, with its latent error‐free counterpart, $M_{\infty }^{k*}\left(t\right)$, in the hazard model. This extension leads to the following measurement error joint model: 


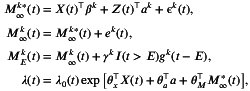


for k=1,2,…,K, where $M_{\infty }^{k*}\left(t\right)$ denotes the latent error‐free value of the kth biomarker at time t before disease onset. The observed biomarker processes $M_{\infty }^{k}\left(t\right)$ and $M_{E}^{k}\left(t\right)$, before and after disease onset, respectively, are assumed to equal the latent biomarker plus an independent measurement error, $e^{k}\left(t\right)$, which is typically assumed to follow a normal distribution. The survival submodel is linked to the latent error‐free biomarker trajectory rather than the observed biomarker process. This extension would allow the association between the longitudinal and survival processes to be driven by the underlying biomarker trajectory rather than by measurement error. Estimating this extended model requires identification of the measurement error variance so that the true biomarker variation can be separated from measurement error. This information may be obtained from replicate measurements, validation studies, or external data providing estimates of the measurement error variance.

In this study, we assumed that the change point in the longitudinal biomarkers coincides with the disease onset time. This assumption is clinically meaningful and appropriate for studying disease progression in individuals with prodromal HD. In more general settings, however, the change point may vary across diseased individuals and need not occur exactly at disease onset. To evaluate the impact of this assumption, we conducted a sensitivity analysis by modifying the simulation study to allow the change point to deviate randomly from the disease onset time. Specifically, we assumed that the change point was given by T=E+d, where E denotes the onset time and d the random perturbation that follows the normal distribution of $N\left(0,\sigma_{d}^{2}\right)$. We considered two scenarios with $\sigma_{d}^{2}=0.25$ and 1, respectively. The proposed method remained robust under the moderate heterogeneity scenario ($\sigma_{d}^{2}=0.25$) with the estimation and statistical inference largely unaffected. However, when heterogeneity became substantial ($\sigma_{d}^{2}=1$), the estimation accuracy and inferential performance deteriorated noticeably. These findings suggest that substantial heterogeneity in the change point should be explicitly modeled rather than ignored. A natural extension is to introduce a latent subject‐specific change‐point component into the likelihood to account for the discrepancy between the disease onset time and the actual change point. Although technically feasible, this extension would require additional methodological development and increased computational effort.

Although the proposed model provides a flexible framework for characterizing HD disease progression with a change point induced by an unobserved milestone disease event, its computational complexity may pose a practical challenge for routine implementation. To address this challenge, we employed an adaptive Fisher scoring algorithm together with several numerical approximations for differentiation and integration, which appeared to be numerically stable (only one run in the 100 bootstrap samples for PREDICT‐HD data had a convergence issue); nevertheless, the computational burden remained substantial. For example, fitting the proposed model to the PREDICT‐HD data required approximately 20 min on our computing platform, which has two Intel Icelake CPUs (56 cores) with 256 GB RAM per node. Developing more efficient numerical algorithms for this class of joint models remains an important direction for future research.

## Funding

This work was supported by DHHS/NIH/NIGMS, Grant/Award Number: U54GM115458.

## Conflicts of Interest

The authors declare no conflicts of interest.

## Data Availability

The PREDICT‐HD data are publicly available in dbGaP PREDICT‐HD Huntington's Disease Study (nindsdac@mail.nih.gov). The simulation and application code can be found in GitHub.
